# Peripersonal Space from a multisensory perspective: the distinct effect of the visual and tactile components of Visuo-Tactile stimuli

**DOI:** 10.1007/s00221-022-06324-8

**Published:** 2022-02-18

**Authors:** Maddalena Beccherle, Stefania Facchetti, Francesca Villani, Marzia Zanini, Michele Scandola

**Affiliations:** 1 Social and Cognitive Neuroscience Laboratory, Department of Psychology, University “La Sapienza” of Rome, Rome, Italy; 2 NPSY-Lab.VR, Department of Human Sciences, University of Verona, Verona, Italy

**Keywords:** Peripersonal space, Multisensory integration, Bayesian methodologies, Visual stimuli, Tactile stimuli

## Abstract

Peripersonal Space (PPS) is defined as the space close to the body where all interactions between the individual and the environment take place. Behavioural experiments on PPS exploit multisensory integration, using Multisensory Visuo-Tactile stimuli (MVT), whose visual and tactile components target the same body part (i.e. the face, the hand, the foot). However, the effects of visual and tactile stimuli targeting different body parts on PPS representation are unknown, and the relationship with the RTs for Tactile-Only stimuli is unclear. In this study, we addressed two research questions: (1) if the MVT-RTs are independent of Tactile-Only-RTs and if the latter is influenced by time-dependency effects, and (2) if PPS estimations derived from MVT-RTs depend on the location of the Visual or Tactile component of MVTs. We studied 40 right-handed participants, manipulating the body location (right hand, cheek or foot) and the distance of administration. Visual and Tactile components targeted different or the same body parts and were delivered respectively at five distances. RTs to Tactile-Only trials showed a non-monotonic trend, depending on the delay of stimulus administration. Moreover, RTs to Multisensory Visuo-Tactile trials were found to be dependent on the Distance and location of the Visual component of the stimulus. In conclusion, our results show that Tactile-Only RTs should be removed from Visuo-Tactile RTs and that the Visual and Tactile components of Visuo-Tactile stimuli do not necessarily have to target the same body part. These results have a relevant impact on the study of PPS representations, providing new important methodological information.

**Supplementary Information:**

The online version contains supplementary material available at 10.1007/s00221-022-06324-8.

## Introduction

The definition of Peri-Personal Space (PPS) changed over time, from the very first definitions strictly connected with the first pieces of evidence from single-neuron recordings (Fogassi et al. 1996; Gentilucci et al. 1983; Graziano et al. 1999, 1994; Rizzolatti et al. 1997, 1981), to the mounting behavioural and electrophysiological shreds of evidence in humans (Bufacchi and Iannetti 2018; Hunley and Lourenco 2018; Serino 2019).

The most recent definitions agree considering the PPS representation being a continuous space closely surrounding the body, somato-topically organized (Farnè and Làdavas 2000; Hyvarinen and Poranen 1974; Robinson and Burton 1980; Robinson et al. 1978; Schicke et al. 2009; Serino et al. 2015; Stone et al. 2017), in which reaching objects and interacting with the environment is possible without locomotion (Borghi and Cimatti 2010; Bufacchi and Iannetti 2018; di Pellegrino and Làdavas 2015; Serino 2019).

The study of PPS mainly relies on behavioural paradigms usually based on the administration of visual- or audio-tactile stimuli on the same body part, using the tactile stimulus as target and the visual or audio component as irrelevant stimulus or distractor.

An example is represented by the Multisensory Interaction Task (Serino 2019; Serino et al. 2007). In this paradigm, participants are asked to respond as fast as possible to a tactile stimulation applied to a body part, while task-irrelevant stimuli (audio or visual) approach the same body part. The Multisensory Reaction Times (RTs) recorded in response to multisensory trials (visual- or audio-tactile trials) are faster when the irrelevant component of stimuli (the Visual or Audio component) is closer to the body part targeted by the tactile stimulus.

Multisensory RTs are then corrected by subtracting the average RTs related to tactile stimulation only (Tactile-Only trials), which is usually presented with delays that do not overlap with any of the Visual- or Audio-Tactile multisensory stimuli (e.g. delays for multisensory stimuli: 1300, 1800, 2500, 3200, 3700 ms; delays for tactile stimuli: 300, 4600 ms; Canzoneri et al. 2012). Other procedures correct the RTs to Multisensory trials subtracting the mean of the condition with Tactile-Only trials with the fastest RT (Serino et al. 2015; Noel et al. 2015a, b). According to the abovementioned procedures of RTs correction, the fundamental assumption on which this task relies is that RTs to Tactile-Only stimuli are considered constant along time and that nor time-related effects, neither inference about the probability of receiving a tactile stimulation modulate Tactile-Only RTs.

In other cases, the RTs to Multisensory Trials are corrected using the mean value of the RTs of Tactile-Only trials applied at different time delays that were matching the delays in the elicitation of the tactile stimulus in the Multisensory Trials (Pfeiffer et al. 2018; Masson et al. 2021).

The implicit assumption of this latter procedure is that Tactile-Only RTs are not constant across time. If Tactile-Only RTs are not constant across time but change across time, it means that the performance in Visuo-Tactile trials is a function of – at least – two different components: the performance in perceiving a tactile stimulus within a precise time window, and the performance modulated by the PPS representation. Therefore, to have an evaluation of the PPS representation, we should remove the Tactile-Only component.

By means of this paradigm, specific somatotopic PPS boundaries have been estimated for hands, face, and trunk tactile stimulation.

Typically, the visual and tactile components of the Visuo-Tactile stimuli are aimed at the same body part, with the purpose of exploiting the characteristics of bi- or tri-modal neurons (Fogassi et al. 1996; Gentilucci et al. 1983; Graziano et al. 1994, 1999; Rizzolatti et al. 1997, 1981). According to researchers’ expectations, behavioural effects observed in the multisensory RTs mimic the properties of bi- or tri-modal neurons seen in single-cell studies. Despite a large amount of literature supporting these hypotheses, there are also some preliminary pieces of evidence suggesting that the Visual and Tactile components of the Visuo-Tactile stimuli do not necessarily have to target the same body part (Scandola et al. 2016, 2020; Schicke et al. 2009). By applying the tactile stimulation to the hand and the visual task-irrelevant stimulation to the feet, these studies showed that visual distractors applied near one body part influence participants’ judgments to tactile stimuli delivered to another body part, therefore, demonstrating an interaction between PPS representations of different body parts.

However, it remains unclear if these behavioural effects on PPS representations using the Multisensory Integration paradigm necessarily require the Visual and the Tactile components of the Visuo-Tactile stimuli to be aimed at the same body part, as no specific investigation has been carried out.

Therefore, this study aims at answering these two research questions: 

1- are Tactile-Only RTs really constant, as considered according to the literature, or can they vary along time, from the starting of a trial until its end?

2- is the performance in detecting tactile stimuli within PPS modulated by the bodily part target of the tactile stimuli, by the bodily part target of the visual stimuli, or by both of them?

On one side, if the target of the tactile stimuli modulates the performance (with no differences when changing the target of the visual stimuli) the PPS representation is somatotopically organised at the tactile level and integrated at the visual one.

On the other side, if the performance is modulated only by the target of the visual stimuli (with no differences when changing the target of the tactile stimuli), the PPS representation is somatotopically organised at the visual sensorial level, and integrated at the tactile sensorial level.

Conversely, if the performance is modulated by both the bodily part target of the tactile stimuli and the bodily part target of the visual stimuli, that probably means that the performance reflects a strong somatotopic organisation, where both visual and tactile stimuli should target the same body part to have an estimation of the PPS representation surrounding that body part.

Finally, if neither the targets of the visual or tactile stimuli modulate the performance, but only the distance is able to explain the modulation, the PPS representation should be integrated suggesting a unique, whole-body PPS representation.

To answer these two research questions, we devised a Visuo-Tactile interaction paradigm. Participants were presented with Tactile-Only trials, Visuo-Tactile (multisensory) trials, and catch trials and asked to detect the tactile stimulations as fast as possible. The beginning of each trial was anticipated by a sound. Tactile-Only trials were administered with the same delays of multisensory stimuli. Furthermore, to investigate whether the body part targeted by the stimulation exerts an influence on PPS representations, the tactile and visual stimuli were administered on three different body locations (i.e., the right cheek, the right hand, and the right foot).

## Material and methods

### Participants

The a-priori sample size was determined using R (R Core Team 2020) and the function ANOVA.Repeat.Measure in the package TrialSize (Zhang et al. 2013), with a power (1-β) of 90%, first type error (α) of 5%, a delta of 10 ms (a-priori meaningful difference, namely in this sample size computation we consider as relevant a difference between RTs of at least 10 ms), the sum of the variance components of 10 ms, and 36 Bonferroni adjustments of interest (the parameter of the function “m”). The suggested sample size was 40.

We collected data from 45 participants, all with normal or corrected-to-normal vision. Three of them were excluded as they resulted to be left-handed according to the Edinburgh Handedness Inventory (Oldfield 1971), because in this experimental work the stimuli were presented on the right side. Two participants were further excluded for technical failures. The final sample was of 40 subjects (19 females, mean ± SD age = 25.8 ± 10.01; 21 males, mean ± SD age = 26.7 ± 10.02). All participants were Italian mother-tongues.

The 40 participants were extremely accurate in the task. Indeed, the lower accuracy was 98.7%, and 34 participants out of 40 reached a 100% accuracy.”

Participants were informed about the experimental procedure and signed the relevant consent form. The study was approved by the Ethics committee of the Province of Verona (Prot. N. 40,378) and was conducted following the ethical standards of the 2013 Declaration of Helsinki.

### Materials

To test PPS, a homemade apparatus was developed to replicate the multisensory interaction task (Serino 2019; Serino et al. 2007). We used an ASUS X53s notebook and the experiment was programmed in OpenSesame ver. 3.2 (Mathôt et al. 2012). We used a lapel microphone for pc to collect vocal RTs to the presence of stimuli, computer headphones to emit white noise to participants, Pico Vibe 5 mm Vibration Motor (Precision Microdrives—www.precisionmicrodrives.com) to administer tactile target stimuli, a 2 m-long LED strip (LED RGB Strip APA102) for the irrelevant visual stimuli and an Arduino Uno R3 (www.arduino.cc) as a microcontroller.

The Arduino Uno R3 was programmed by means of a homemade script in Processing (www.processing.org).

The OpenSesame program counterbalanced conditions across participants, randomized trials, sent the type of the trail to the Arduino Uno R3, emitted the sound for the beginning of a new trial and the white noise, and recorded the RTs. The Arduino Uno R3 converted the signal from the OpenSesame program to the correct combination of Visuo-Tactile stimuli. To obtain the illusion that a group of 4 LEDs was approaching the participant at a velocity of about 32 cm/sec, when the LED Strip received the signal from the Arduino Uno R3, it serially lighted up and switched off LEDs in groups of 4 white LEDs. Once the vibration device received the signal from the Arduino Uno R3 microcontroller, it vibrated for 100 ms. Moreover, the Arduino Uno R3 was connected to the notebook to precisely signal the beginning of the trial.

The LED RGB Strip (APA102) was glued on a wooden bar placed in front of the participant, near the right hand, the right part of the face, or the right foot, according to the experimental condition (for a similar set-up using LEDs, see Noel et al. 2020 Experiment 2).

The vibration device was placed on the right hand, on the right cheek, or on the right foot according to the experimental condition.

### Procedure

Participants were seated on a comfortable, height-adjustable chair, adjusted to place the LED Strip at the level of the chin, avoiding any other additional adjustment during the experimental session or uncomfortable position for the participants.

They signed the consent form and filled out the Edinburgh Handedness Inventory (Oldfield 1971).

Then, the experimental task was explained, and participants wore the headphones and the lapel microphone as near as possible to their mouth.

**Fig. 1:**
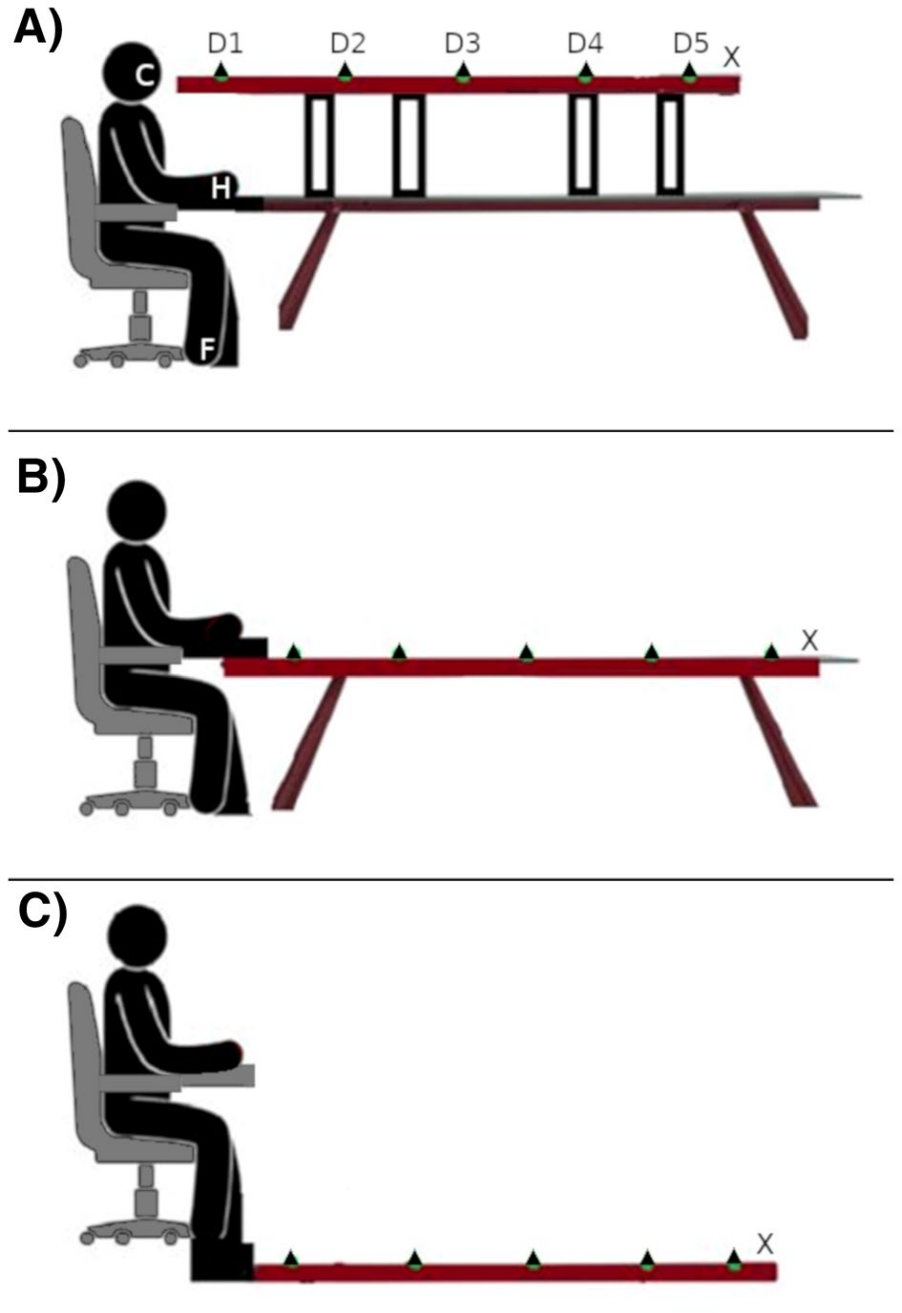
Graphical representation of the different experimental conditions. The brown bar with the triangles represents the LED Strip, and the triangles the positions of the visual irrelevant stimuli when the tactile stimuli were administered. The distances for the visual stimuli and the delays for the Tactile-Only stimuli were: D1 = 1 cm/6.125 s; D2 = 49 cm/4.564 s; D3 = 98 cm/3.063 s; D4 = 147 cm/1.531 s; D5 = 196 cm/0 s. The X was the initial fixation point, 2 m away from the participant. **A** Position of the LED Strip for the Face level of the Light Location factor. C, H and F represent the three levels of the Tactile Location factor respectively, i.e. (right cheek) Face, (right) Hand and (right) Foot. In this figure, we report only this combination, but the three Tactile Location levels interacted with all Light Location levels. **B** Hand level of the Light Location factor. **C** Foot level of the Light Location factor

Before the beginning of each trial, the white noise was administered through the headphones to signal the upcoming presentation of a new trial, and participants were instructed to direct their gaze to the fixation point (see Fig. 1).

After a random delay of 100–300 ms, the trial started, and the visual stimuli appeared approaching the participant. They had to verbally answer as soon as possible when they felt the tactile stimulation on their body. We chose the non-word “TOH” as the answer word because it does not contain fricative consonants and thus allows a better RTs determination (Scandola et al. 2016, 2020).The experimental design was a repeated-measure design, with the following factors: Light Location (at the elevation of the Face, or of the Hand, or of the Foot), Tactile Location (placed on the Face, or on the Hand, or on the Foot) and Distance (D1 = 1 cm / 6.125 s; D2 = 49 cm / 4.564 s; D3 = 98 cm / 3.063 s; D4 = 147 cm / 1.531 s; D5 = 196 cm / 0 s).

The Light Location factor corresponded to the position of the LED strip, whose elevation was adjusted to match the right side of the face height (Fig. 1A), or the right-hand height (Fig. 1B), or the right foot height (Fig. 1C). The Tactile Location factor corresponded to the position of the vibration device on the participants, i.e. right cheek (halfway between the ear and the mouth), right thumb and right hallux (see Fig. 1A). The Distance factor represented respectively: the position of the visual stimuli on the led strip at which the tactile stimulation was delivered in Visuo-tactile trials and the delay between the starting of the white noise and the administration of the tactile stimulus in Tactile-Only trials.

The 9 conditions resulting from the interaction of the Light Location factor and the Tactile Location factor were counterbalanced across participants.

Each condition consisted of three different typologies of trials: visuo-tactile trials (30 trials, 6 repetitions each distance); Tactile-Only trials (15 trials, 3 repetitions each distance) and visual-only catch trials (3 trials) to test whether the participants answered to imagined tactile sensations or to proper tactile sensations. A total of 48 trials per condition were administered in a randomized order.

After each condition participants could take a break of up to 5 min each. The whole experiment lasted about 1.5 h.

### Data handling and Statistical procedure

The RTs of the trials where participants did not answer, or outside the range computed for each participant by means of the Interquartile Range method (${Q}_{1}-\frac{2}{3}\bullet IQR ; {Q}_{3}+\frac{2}{3}\bullet IQR$)^1^ were removed. For each participant, RTs were then aggregated by computing their means for the Typology of the trial (Visuo-Tactile, Tactile-Only, and Catch trials), Distance, Light and Tactile Location conditions.

Furthermore, we computed an additional data set with normalized RTs, by subtracting the RTs related to Tactile-Only trials from the RTs of the Visuo-Tactile trails for each participant and each combination of factors (Distance, Light Location and Tactile Location).

The statistical analyses were performed with R ver. 4.0.0 (R Core Team 2020) and JAGS (Plummer 2017), the R packages jagsUI (Kellner 2019), coda (Plummer et al. 2006) and mcmcse (Flegal et al. 2020). The JAGS codes for Bayesian Models are reported in the Supplementary Materials (SM1).

Data Analyses were executed within the Bayesian framework (de Laplace 1825; Kruschke 2014; Wagenmakers 2007) with Hierarchical Linear Models (Gelman and Hill 2006; Pinheiro and Bates 2000). The model selection was executed by means of the Product Space Method (Carlin and Chib 1995; Gamerman and Lopes 2006, p. 257; Lodewyckx et al. 2011) and the Indicator Variable Selection method (IVS, Kuo and Mallick 1998; for examples see Scandola et al. 2020, 2019). These two methods are similar: in the Product Space Method, a series of models representing various hypotheses are tested. Using a categorical index hyperprior encompassing the models, we obtain the number of times the models were visited to account for the observed data. These proportions are indicated by P(H_id_|D), with *id* being the hypothesis identifier. These values range from 0 to 1 (maximum probability), providing an intuitive measure of which hypothesis is more trustworthy. In the Indicator Variable Selection method each factor of the model is selected by means of a Bernoulli hyperprior (1 = selected; 0 = unselected). By averaging the Bernoulli hyperpriors, we obtain the proportion of times the factor was selected to describe the data. These proportions can range between 0 and 1 and are indicated by IVS.

IVS and P(H_id_|D) support the alternative hypothesis very strongly when > 0.99, strongly when > 0.95, and positively when > 0.75 (Lodewyckx et al. 2011; Raftery 1995).

Conversely, IVS and P(H_id_|D) values < 0.01, < 0.05, < 0.25 respectively represent very strong, strong and positive supporting evidence for the null hypothesis. All IVS and P(H_id_|D) values within the 0.25 ~ 0.75 range are inconclusive, being unable to support neither alternative nor the null hypothesis, and applying the Occam’s razor principle, will not be considered.

The same analyses were computed also with frequentist statistics by means of ANOVAs with the afex package (Singmann et al. 2017), obtaining similar results, reported in SM2.

Data analyses were organized in three consecutive steps.

The aim of the first one was to answer our first research question: are the baseline Tactile-Only RTs really constant, or can they vary over time, from the starting of a trial until its end?

Therefore, we used the Product Space Moment method: the categorical hyperprior could, with the same prior probability, choose among 3 different hypotheses:^2^

H_0_ = there are no differences between Tactile-Only and Visuo-Tactile RTs;

$${\beta }_{i}\sim N\left(0,{\sigma }_{i}\right)$$

$${\xi }_{1\dots j}\sim \mathrm{MN}\left(0,\Omega \right)$$

$${\mathrm{RT}}_{\mathrm{Visuo}-\mathrm{Tactile}}=X\times \beta +Z\times \xi + \epsilon$$

$${\mathrm{RT}}_{\mathrm{Tactile}-\mathrm{Only}}=X\times \beta +Z\times \xi + \epsilon$$

In this formula, ${\beta }_{i}$ represents the coefficients for the fixed effects, ${\sigma }_{i}$ are the standard deviations for the fixed effects, ${\xi }_{j}$ are the coefficients for the random effects, $\Omega$ is the variance–covariance matrix for the random effects, $\epsilon$ is the error term, *X* is the contrast matrix of the fixed effects, *Z* the contrast matrix for the random effects, *N* denotes the normal distribution and MN the multivariate normal distribution. As it is possible to notice, the formula for the Visuo-Tactile RTs is the same for the Tactile-Only RTs.

H_1_ = there are differences between Tactile-Only and Visuo-Tactile RTs, and Tactile-Only RTs are constant;

$${\beta }_{i}\sim N\left(0,{\sigma }_{i}\right)$$

$$\tau \sim N\left(0,\varsigma \right)$$

$${\xi }_{1\dots j}\sim MN\left(0,\Omega \right)$$

$${vRT}_{\mathrm{Visuo}-\mathrm{Tactile}}=X\times \beta +Z\times \xi + \epsilon$$

$${vRT}_{\mathrm{Tactile}-\mathrm{Only}}=\tau + \epsilon$$

$\tau$ is the constant value for Tactile-Only RTs and $\varsigma$ its standard deviation. In this case, the formulas for the Visuo-Tactile and Tactile-Only RTs are different, but the Tactile-Only RTs are constant.

H_2_ = there are differences between Tactile-Only and Visuo-Tactile RTs, and Tactile-Only RTs are not constant over time (i.e., there can be a variation of Tactile-Only RTs over the time, from the beginning to the end of the trials).

$${\beta }_{i}\sim N\left(0,{\sigma }_{i}\right)$$

$${\tau }_{j}\sim N\left(0,{\varsigma }_{j}\right)$$

$${\xi }_{1\dots j}\sim MN\left(0,\Omega \right)$$

$${vRT}_{\mathrm{Visuo}-\mathrm{Tactile}}=X\times \beta +Z\times \xi + \epsilon$$

$${vRT}_{\mathrm{Tactile}-\mathrm{Only}}=X \times \tau +Z\times \xi + \epsilon$$

In this formulation, we can notice that for both the Visuo-Tactile and Tactile-Only RTs we have different and complete hierarchical linear models.

If the data supports the H_0_ hypothesis, thus the experimental procedure may not be effective in capturing the PPS representation, showing instead an effect that is reducible to timing effects on tactile perception.

If the H_1_ hypothesis is true, then the Visuo-Tactile trials may be able to elicit PPS effects almost independently from Tactile-Only trials (assuming that Tactile-Only RTs are constant, the subtraction of a constant value to all the Visuo-Tactile trials does not change the differences between the Visuo-Tactile trials at different distances).

If H_2_ is true, the Visuo-Tactile trials may be modulated by PPS representation and effects of tactile sensations expectancy. This means that even if Visuo-Tactile trials can give an estimation of PPS representation, within them there also is an effect of the expectancy of tactile sensations that change over time, and that this modification can follow non-linear trends. However, in this case, it would be necessary to clean the RTs of Visuo-Tactile trials from RTs of Tactile-Only trials, because they could be influenced by Tactile-Only RTs variation.

The second step of the analyses aimed at identifying which factor would account for Tactile-Only RTs variation. Therefore, by means of IVS, we tested the Tactile-Only RTs with Distance, Light and Tactile Location factors as fixed effects, and Light and Tactile Location as random effects. In Tactile-Only trials, the Distance factor is represented by the delay between the starting of the white noise and the administration of the tactile stimulus.

The last step focused on the second research question: is the somatotopic PPS organization modulated by the bodily part target of the tactile stimuli, or by the bodily part target of the visual stimuli, or by both of them?

To answer this question, normalised Visuo-Tactile RTs were analysed using IVS with Distance, Light and Tactile Location as fixed effects, and Light and Tactile Location as random effects.

## Results

### Catch trials

Only 3 participants answered to some catch trials, with a maximum frequency of 2 trials out of 27 (7.4%). Therefore, all participants understood and correctly performed the task, and no other subjects had to be further removed from the analyses.

### Test of the three hypotheses

We first analysed the three hypotheses H_0_, H_1_ and H_2_. PSM showed that the RTs of the Tactile-Only trials were different from the ones of the Visuo-Tactile trials [P(H0|D) = 0] and that they were not constant [P(H_1_|D) = 0, P(H_2_|D) = 1]. The mean (sd) for Tactile-Only trials was 374.04 (125.43) ms, while for Visuo-Tactile trials was 354.31 (125.46) ms.

Therefore, we observed that Visuo-Tactile trials may be able to capture PPS effects, but also that they are influenced by Tactile-Only trials timing effects.

However, to better understand the effects of this result, the second and the third step of analyses are necessary.

### Analyses of tactile-only trials

According to the second step of the analyses, Tactile-Only RTs were influenced only by the Distance [IVS = 1, mode (95% Highest Density Interval) 1 cm = 374.98 (370.53, 379.05) ms; 49 cm = 378.14 (373.63, 382.48) ms; 98 cm = 403.29 (397.12, 408.87) ms; 147 cm = 375.87 (369.22, 384.67) ms; 196 cm = 389.09 (380.43, 394.73) ms], whereas all the other factors and interactions supported the null hypothesis (all IVS < 0.13). The only exception was represented by the Tactile location (IVS = 0.535) and Distance:Tactile Location interaction (IVS = 0.333) whose contributions were however inconclusive, and applying the Occam’s razor principle not further considered.

These effects show that Tactile-Only RTs are modulated by time, and these effects should be removed from RTs when analysing PPS representations.

**Fig. 2:**
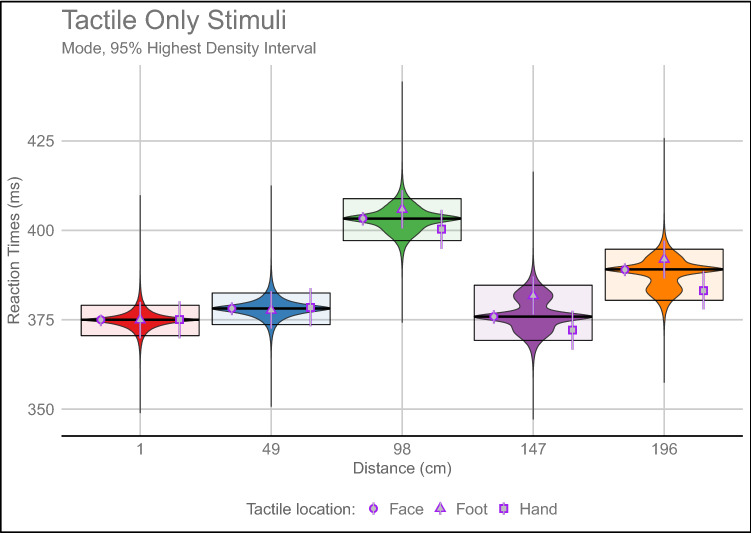
Posterior distributions of the Tactile-Only RTs Bayesian Multilevel Linear model. Violin plots represent the distributions. The bold line in the middle of the box is the mode, and the upper and lower boundaries of the box represent the 95% Highest Density Interval (HDI). The curves are probability density curves represented along the y-axis instead of the *x* axis, plotted on each side. The grey points, encircled by purple points, represent the mode of the posterior distributions for the different tactile locations. The grey/purple errorbars are the 95% HDI. In the *x* axis are reported the Distances used for the Visuo-Tactile trails, that corresponds to the specific temporal delays used for Tactile-Only trials: 1 cm/6.125 s; 49 cm/4.564 s; 98 cm/3.063 s; 147 cm/1.531 s; 196 cm/0 s.

See Fig. 2 for a graphical representation of the posterior distributions of Tactile-Only RTs.

### Analyses of Visuo-Tactile trials

**Fig. 3:**
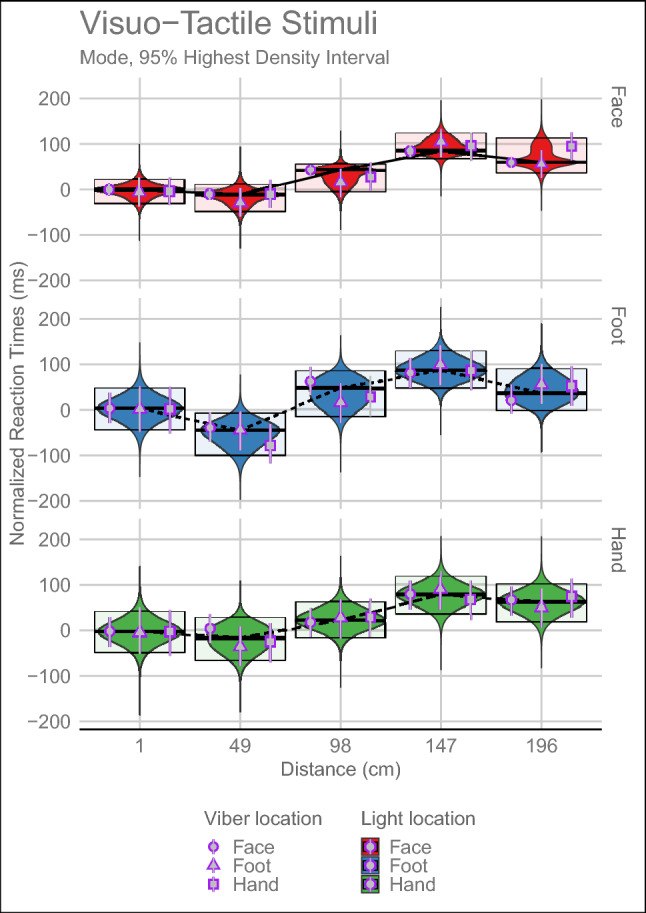
Posterior distributions of the Visuo-Tactile normalized RTs Bayesian Multilevel Linear model. In the x-axis are reported the Distances along the LED strip at which the tactile stimulation was delivered: 1 cm/6.125 s; 49 cm/4.564 s; 98 cm/3.063 s; 147 cm/1.531 s; 196 cm/0 s. The three different panels represent the three different Light Locations, while the grey points, encircled by purple points, represent the mode of the posterior distributions for the different tactile locations. For further details, please see the caption of Fig. 2.

Normalised RTs showed that the alternative hypothesis was true for the Light Location:Distance interaction (IVS = 0.980), Light Location (IVS = 0.986) and Distance (IVS = 1). Tactile Location and its interaction with Distance resulted inconclusive (IVS = 0.638 and 0.604 respectively). Therefore, applying Occam’s razor principle, they will not be further analysed. All the other interactions supported the null hypothesis (all IVS = 0). See Fig. 3 for a graphical representation of the posterior distributions of Visuo-Tactile trials, and Table 1 for the estimates of the posterior distribution.

**Table 1:** Mode and 95% HDI of the Posterior Distribution for each distance and Light Location

Distance (cm)	Face	Foot	Hand
1	− 0.98 [9.77, − 12.09]	2.22 [35.34, − 27.71]	− 3.3 [26.4, − 35.4]
49	− 10.68 [0.12, − 22.69]	− 39.04 [− 6.73, − 70.07]	2.77 [35.57, − 26.53]
98	42.56 [53.86, 31.06]	61.12 [94.46, 31.14]	16.35 [47.75, − 14.33]
147	83.51 [94.42, 71.62]	80.07 [112.62, 49.27]	78.26 [107.93, 45.8]
196	59.11 [70.42, 47.63]	21.33 [54.81, − 8.54]	65.76 [94.31, 32.23]

Furthermore, to answer our second research question (investigate whether the somatotopic PPS organization is modulated by the body part targeted by the visual stimuli, or by the tactile stimuli or by both), we contrasted the normalized RTs at each distance against the next distance divided by Light Location, to verify if there were differences in PPS boundaries.

When the Light Location was at the Foot-level, the PPS boundary was between 98 and 147 cm (IVS = 0.799), whereas when it was at the Face-level and at the Hand-level, the PPS boundary ranged between 49 and 98 cm (IVS = 0.757 and 0.839, respectively).

## Discussion

This work addressed two main research questions: (1) whether Tactile-Only RTs are constant over time, as considered according to the literature, or if they vary, from the beginning of a trial until its end; (2) whether the somatotopic PPS organization is modulated by the bodily part targeted by the tactile stimuli, by the visual stimuli, or by both of them.

We directly analysed three specific hypotheses: H_0_ = there are no differences between Tactile-Only and Visuo-Tactile RTs, with the consequence that Visuo-Tactile RTs are not actually modulated by PPS representation; H_1_ = there are differences between Tactile-Only and Visuo-Tactile RTs, and Tactile-Only RTs are constant, namely the Visuo-Tactile RTs are modulated by PPS representation independently from the Tactile-Only RTs, that are constant and, therefore, irrelevant; H_2_ = there are differences between Tactile-Only and Visuo-Tactile RTs, and Tactile-Only RTs are not constant over time, with the consequence that Visuo-Tactile RTs are modulated by both PPS representation and effects of tactile expectancy.

Two main results have emerged from this work, showing the validity of hypothesis H_2_: (a) Tactile-Only stimuli impact the participants’ performance in a non-linear fashion, determining higher RTs around the central part of the PPS curve; and (b) normalized RTs of multisensory Visuo-Tactile stimuli are modulated by the Light Location factor.

These results have important implications in behavioural PPS studies and may also provide new useful insights for PPS theories.

### The neglected impact of the unimodal tactile sensory system on the Multisensory PPS representation

To the best of our knowledge, this study explicitly demonstrates for the first time, in the field of multisensory PPS that RTs to unimodal Tactile-Only stimuli are neither constant nor following a linear monotonic trend (a non-linear, but monotonic trend was instead found in Hobeika et al. 2020).

Taking into consideration this new evidence when studying PPS is of crucial importance, because it may greatly affect its behavioural estimation.

Indeed, as hypothesised in the Introduction, the RTs of Visuo-Tactile trials might be the effect of two co-occurring phenomena: the modulation of PPS representation on the performance, and the modulation of the conscious waiting for a tactile stimulus within a specific and well determined time window. To have a better estimation of the influence of PPS representation on the performance, the Tactile-Only baseline should not be computed only as a unique value but should be estimated for each delay, representing a different visual distance.

Previous studies tried to estimate the effects of tactile expectation on PPS.

Kandula et al. (2017) manipulated the ratios between Visuo-Tactile trials and Catch trials, according to the following odds: 1:1, 4:3, 2:1, 4:1, to investigate the influence of tactile expectations (i.e. the expectation of receiving a tactile stimulation) on participants’ behavioural performance.

They applied computational models, combining the ratios between Visuo-Tactile and Catch trials and a simple linear function describing the intra-trial tactile expectation level, constantly increasing from 0% (at the beginning of the trial) to 100% (at the end of the trial).

According to these computational models, only when the ratio between Visuo-Tactile and Catch trials was 4:1 or 2:1, the task fitted a generalized sigmoid function. This result was then supported and corroborated by an experimental application with real participants (Kandula et al. 2017).

Despite the undeniable interest and importance of these findings, they, however, consider the intra-trail expectation level for Tactile-Only stimuli as a linear monotonic function.

Recently, a further study found that unimodal Tactile-Only RTs follow a non-linear pattern (Hobeika et al. 2020). In this case, however, unlike in the work by Kandula et al. (2017), the participants’ performance was described by a logarithmic monotonic function: a difference possibly due to the experimental paradigm. In this study, in fact, they used looming audio-tactile stimuli as multisensory stimuli and administered constant audio stimuli during Tactile-Only trials. The use of audio stimuli during both the multisensory and Tactile-Only trials may have shaped participants’ expectancies.

Our results show that Tactile-Only RTs are distributed along a bell-shaped curve (see Fig. 2), independently of Light and Tactile Locations. This pattern of responses is observable also in studies from other groups investigating PPS, even if in these works, they do not report any space-dependent relation between the Tactile-Only trials and Distance (Noel et al. 2020, Fig. 1A; Serino et al. 2018, Fig. 4A). This difference could be due to the difficulty of capturing non-monotonic effects using traditional linear models.

A possible explanation for the fastest RTs to the earlier and the later Tactile-Only trials (the “bell-shaped” curve distribution) could be the co-occurrence of two different phenomena. The first phenomenon is a simple attentional effect: the detection of a stimulus, if alerted by a sound preceding the stimulus within 100 ms and 2 s, will be fastened (Luce 1991, p. 77). D5 and D4 are both within 2 s, while D3 is at 3063 ms, outside the window that can benefit from the sound onset. The second effect is given by the cumulated subjective expectation about the probability of receiving the Tactile-Only stimulation on simple RTs (Gordon 1967). In this case, the participants, that after a short number of trials will implicitly understand the maximum duration of trials, will be aware when they are close to the end of the trial, and therefore, the expectancy of being elicited with a tactile stimulus will increase. The combination of these two effects can explain the shape of RTs to Tactile-Only stimuli: D5 and D4 benefit from the attentional boost caused by the sound indicating the start of the trial, while the detection in D1 and D2 is improved by the cumulated subjective probability of being elicited with a tactile stimulus.

However, other aspects can have an influence on Visuo-Tactile trials. For example, gravitational effects may influence sensory perception (Peru et al. 2006), as well as the position of the Visual and Tactile devices of stimulation (for an extensive experimental overview, see Marini et al. 2017), leading to further biases in Multisensory RTs that should be taken into consideration when studying PPS in different body and stimuli positions.

Another important source of bias is the serial effects of Tactile-Only stimuli on Multisensory Visuo-Tactile stimuli. Indeed, a previous Tactile-Only stimulus, delivered at a specific delay, changes the expectations about the following multisensory Visuo-Tactile stimulus: the expectation about the stimulus presentation is determined and based on the timing of stimulus administration in the previous trial (i.e., on the delay between the beginning of the trial and the stimulus administration) (Noel et al. 2020). This interpretation is in line with classical psychophysical findings, according to which the perception of the visual and tactile part of a stimulus, administered at different delays, is shifted towards their mean delay (Kuschel et al. 2010; Scandola et al. 2012) and with findings on the serial dependence of stimuli, according to which subjects typically err toward the previous stimulus (Cicchini et al. 2017; Fernberger 1920; Fischer and Whitney 2014; Yu and Cohen 2008).

All the above-mentioned phenomena can have a detrimental effect on the investigation of the relationship between Multisensory RTs and PPS estimation in the Multisensory Interaction task. In particular, the “bell-shaped” distribution of Tactile-Only RTs may shift the PPS boundary far from its real location. Furthermore, the position of previous Tactile-Only stimuli and the ratio between Multisensory and Catch-trials may modify the expectancy of receiving a tactile stimulation and, consequently, the related RTs. Fortunately, all these confounding factors can be limited. A possible solution to control for Gravitational effects, “bell-shaped” or other non-linear effects of the Tactile-Only RTs on Multisensory RTs may be the subtraction of Tactile-Only RTs to Multisensory RTs in the same conditions. The ratio between Multisensory and Catch-Trials should be equal or lower to 2:1 (Kandula et al. 2017), and the serial dependencies between Multisensory and Tactile-Only trials can be solved with a rigorous, not biased, trial randomization.

### The relevant importance of the irrelevant visual stimuli

Our experiment shows, for the first time in a systematic and clear way, that the Light Location factor was able to determine the dependence of Normalize RTs on Distance more than the Tactile Location factor, breaking the traditional connection between the visual and tactile sensory systems within the PPS representation.

This finding may seem in contrast with the very first definitions of Multisensory PPS, inspired by single-cell recording studies where bimodal neurons answered to tactile stimulations delivered on a specific body part and to visual stimuli approaching the same area (Cléry et al. 2015; Rizzolatti et al. 1997).

However, this particular typology of neurons is capable of reacting to visual stimuli approaching an area that is larger than the one targeted by tactile stimuli. In Fogassi et al. (1996) 23% of bimodal neurons answered to visual stimuli approaching an area larger than the tactile one.

Behavioural studies on humans already showed some evidence indicating that the correspondence between the location of visual and tactile stimuli is not strictly necessary (Bassolino et al. 2010; Scandola et al. 2016, 2020; Schicke et al. 2009; Serino et al. 2015). Schicke et al. (2009) found that by placing the tactile stimulators on the hands, and visual distractors near the feet, classical PPS effects were observable.

Moreover, a study by Bassolino et al. (2010) on habitual PC users demonstrated that observing the computer monitor, the target of an irrelevant audio stimulus, while a tactile stimulation was delivered on the hand holding the mouse, led to the incorporation of the monitor in participants’ PPS representation.

Further work by Serino et al. (2015, experiment 5) showed that changing the position of the hand and placing it near the trunk modified hand PPS boundaries: the hand PPS enlarged and reached the dimension of the trunk PPS. In this case, even if the tactile stimulation is applied to the hand, it is, however, difficult to disentangle if the observed effect on PPS is due to an effective extension of the hand PPS or to an estimation of the trunk PPS *per se*.

All these works highlight the important role of vision in PPS representation, confirmed also by a seminal study by Pavani et al. (2000). In this study, using the well-known rubber hand paradigm, the authors found that only the congruent condition, represented by the congruency between the position of rubber hands and the participants’ body, elicited PPS effects. Findings by Scandola et al. (2020) as well showed that in healthy participants an incongruent visuo-motor stimulation of lower limbs impaired lower-limb PPS representations.

To sum up, the findings in this article, corroborated by some previous evidence, seem to pinpoint that the PPS representation is determined by the body part that is the target of the visual (or audio) “irrelevant” stimulus, and not from the body part target of the tactile stimulus.

### The theoretical contribution of our results to PPS definition

The first PPS definitions coming from single-cell studies on macaques and neuropsychological studies on neurological patients (Cléry et al. 2015; di Pellegrino and Làdavas 2015), led to theoretical conceptualizations strictly linked to action and defensive functions and characterized by strong coherence between visual and tactile stimuli.

In recent years, PPS theories underwent a significant development thanks to the experimental findings mainly coming from behavioural studies on healthy participants.

These studies demonstrated that PPS is more plastic than shown in single-cell experiments: anxiety and phobia (Longo and Lourenco 2006; Sambo and Iannetti 2013; Taffou and Viaud-Delmon 2014), interoceptive sensations (Ardizzi and Ferri 2018; Scandola et al. 2020), social stimuli (Fini et al. 2015; Gigliotti et al. 2019; Heed et al. 2010; Maister et al. 2015; Teneggi et al. 2013), the economic, appetitive, negative and positive valence of the approaching stimuli (Spaccasassi et al. 2019) are able to modulate PPS in human healthy subjects.

With this wide range of results, it is natural wondering how many PPS may exist (de Vignemont & Iannetti 2014). Nowadays influential theories agree on the existence of a single and continuous PPS leading to different behavioural responses according to the nature of the stimuli (Bufacchi & Iannetti 2018) or to the other systems with which is interacting (Serino 2019).

However, also in these new and more recent conceptualizations, the tactile and the audio or visual stimuli are aimed at the same body part, a heritage from the single-cell studies on bimodal neurons. Our results instead seem to suggest that the somatotopy of PPS representation is mainly modulated by the location of the visual stimuli, while the location of the tactile stimuli, in light of these results, is secondary, if not totally irrelevant.

Further studies are needed to validate this claim.

However, at the moment, these results lead to a further possible question: Are the PPS representations assessed by single-cell studies and the ones emerging from studies on humans the same PPS representations?

This consideration leads to a further possible question: Are the PPS representation assessed by single-cell studies and the ones emerging from studies on humans the same PPS representations?

The strong links and parallelisms are evident, but the behavioural effects of PPS representations are the result of a complex interaction between many and diverse brain networks, such as the network related to body representation (Grivaz et al. 2017) and action (Brozzoli et al. 2011a, b). Despite their utmost importance, single-cell studies show only a minimal part of all these interactions.

Therefore, from a behavioural point of view, PPS is a continuous, body-centred spatial representation, whose estimation does not necessarily require tactile and visual or audio stimuli to be referred to the same bodily part.

## Conclusions

In this work, face- hand- and foot-centred PPS representations were assessed for the first time using an adapted version of the well known Multisensory Integration Task, administrating the Visual and Tactile components of stimuli to these three different body parts.

Our results show that behavioural estimations of PPS representations are influenced by Tactile-Only stimuli. This new evidence provides useful and helpful information to remove potential biases when studying PPS.

Importantly, our results suggest that PPS representations are shaped by the bodily part target of the Visual stimuli.

These results may have potential consequences on methodological aspects of PPS studies, suggesting that for a correct estimation of the PPS representation we should normalize the RTs from Multisensory trials on the RTs from Tactile-Only trials for each distance.

Moreover, these results may have a potential impact on PPS definitions, showing that visual stimuli and tactile stimuli do not necessarily have to target the same body part.

## Supplementary Information

Below is the link to the electronic supplementary material.

Supplementary file1 (DOCX 41 KB)

## Data Availability

Beccherle, M., Facchetti, S., Villani, F., Zanini, M., & Scandola, M. (2020, June 12). Peripersonal Space from a multisensory perspective: the distinct effect of the visual and tactile components of Visuo-Tactile stimuli. Retrieved from osf.io/z3pfa.

## Footnotes

1 Q_1_ and Q_2_ are respectively the first and third quartile; IQR = Interquartile Range.

2 These formulas are incomplete. For a better description see SM1.
