# Supplementary material for: Peripersonal Space from a multisensory perspective: the distinct effect of the visual and tactile components of Visuo-Tactile stimuli
Source: Exp Brain Res. 2022 Feb 18;240(4):1205–17. doi: 10.1007/s00221-022-06324-8 (PMC9015983; doi:10.1007/s00221-022-06324-8)
Supplement: Supplementary file 1 — Supplementary file1 (DOCX 41 KB) [file 221_2022_6324_MOESM1_ESM.docx]

# Supplementary Materials of “*Peripersonal Space from a multisensory perspective: the distinct effect of the visual and tactile components of Visuo-Tactile stimuli*”

Beccherle M., Facchetti S., Villani F., Zanini M., Scandola M.

## SM1 – Bayesian Models Specifications and JAGS codes

### SM1.1 – Model for the analyses of Visuo-Tactile and Tactile-Only Reaction Times with the Product-Space method

This Bayesian model uses a hyper-hyperprior parameter ι, that is distributed along a multinomial distribution with three possible outcomes, all with the same prior probability of 1/3, to encompasses the three different hypotheses.

The three different hypotheses are given by three different Bayesian Hierarchical Models that analyse Visuo-Tactile and Tactile-Only RTs with different constraints.

The three hypotheses are:

H_0_ = there are no differences between Tactile-Only and Visuo-Tactile RTs;

H_1_ = there are differences between Tactile-Only and Visuo-Tactile RTs, and Tactile-Only RTs are constant;

H_2_ = there are differences between Tactile-Only and Visuo-Tactile RTs, and Tactile-Only RTs are not constant along time.

| 1  2  3  4  5  6  7  8  9  10  11  12  13  14  15  16  17  18  19  20  21  22  23  24  25  26  27  28  29  30  31  32  33  34  35  36  37  38  39  40  41  42  43  44  45  46  47  48  49  50  51  52  53  54  55  56 | model **{**  # hyper-hyperprior iota emcompassing the 3 hypotheses  pi **<-** c**(**1**/**3,1**/**3,1**/**3**)**  iota **~** dcat**(**pi**)**  # iota == 1, H0: beta.int = beta.tac  # iota == 2, H1: beta.int <> beta.tac; beta.tac only intercept  # iota == 3, H2: beta.int <> beta.tac    # Define priors for the variance-covariance matrix of the random effects  Omega.u **~** dwish**(**W**[**,**]**, dfwish**)**  Sigma.u **<-** inverse**(**Omega.u**[**,**])**    # Define hyperpriors for random effects  **for(**k **in** 1**:(**dfwish**-**1**)){**  xi_tau**[**k**]** **<-**xi_mu**[**k**]***sqrt**(**Sigma.u**[**k,k**])**  xi_mu**[**k**]** **~** dnorm**(**0, 0.001**)**  **}**    # Define correlations among random effects  **for(**r1 **in** 1**:(**dfwish**-**2**)){**  **for(**r2 **in** **(**r1**+**1**):(**dfwish**-**1**)){**  rho**[**r1,r2**]** **<-** Sigma.u**[**r1,r2**]/**sqrt**(**Sigma.u**[**r1,r1**]***Sigma.u**[**r2,r2**])**  **}**  **}**    # Define priors for the random effects  **for** **(** s **in** 1**:**Nsubjects **)** **{**  u**[**s,1**:(**dfwish**-**1**)]** **~** dmnorm**(**xi_mu**[**1**:(**dfwish**-**1**)]**,Omega.u**[**1**:(**dfwish**-**1**)**,1**:(**dfwish**-**1**)])**  **}**    # population parameters (fixed parameters)  **for** **(** j **in** 1**:**Nparameters **)** **{**  beta**[**j**]~** dnorm**(**0,1**/**s**[**j**])**  tau**[**j**]~** dnorm**(**0,1**/**s**[**j**])**    s**[**j**]** **~** dgamma**(**1,0.001**)**  **}**  sigma **~** dgamma**(**1,0.001**)**  #likelihood  **for** **(**i **in** 1**:**Ntotal.int**)** **{**  mu.int**[**i**]** **<-** inprod**(**XF.int**[**i,**]**,beta**)** **+** inprod**(**XR.int**[**i,**]**, u**[**subj.int**[**i**]**,**])**  RT.int**[**i**]** **~** dnorm**(**mu.int**[**i**]**,1**/**sdscal**)**  **}**    **for** **(**i **in** 1**:**Ntotal.tac**)** **{**  mu.tac**[**i**]** **<-** ifelse**(**iota**==**1,inprod**(**XF.tac**[**i,**]**,beta**)**, #H0  ifelse**(**iota**==**2,tau**[**1**]**, #H1  inprod**(**XF.tac**[**i,**]**,tau**)))** #H2  **+** inprod**(**XR.tac**[**i,**]**, u**[**subj.tac**[**i**]**,**])**  RT.tac**[**i**]** **~** dnorm**(**mu.tac**[**i**]**,1**/**sdscal**)**  **}**  **}** | The hyper-hyperprior ι comes from a Multinomial distribution with equal probabilities each hypothesis.  $\pi_{1\ldots3}=1/3$  $\iota\sim Multinomial(\pi_{1\ldots3})$  The variance-covariance matrix for the random effects comes from the Inverse of a Wishart distribution.  $\Omega\sim Wishart(diag(dfwish-1),dfwish )$  $\Sigma=\Omega^{-1}$  The coefficients of the random parameters are then estimated  $\xi_{1\ldots k}\sim Normal\left( 0,0.001 \right)$  ${sd\xi}_{1\ldots k}=\xi_{1\ldots k}\times\Sigma_{k,k}$  Next, also the correlation values between random parameters are computed  $\rho_{i,j}={\Sigma_{i,j}}/{\sqrt{\Sigma_{i,i}\times\Sigma_{j,j}}}$  Finally, the random effects for each participant is estimated  $u_{s,k}\sim MultiNormal(\xi_{1\ldots k}$,$\Omega_{k,k})$  Then, we estimate the fixed effects parameters for Tactile-Only and Visuo-Tactile RTs  $\sigma_{j}\sim Gamma(1,0.001)$  $\beta_{j}\sim Normal(0,1/{\sigma_{j}})$  $\tau_{j}\sim Normal(0,1/{\sigma_{j}})$  Next, we estimate the variance of the Likelihood distribution  $\sigma\sim Gamma\left( 1,0.001 \right)$  Finally, the likelihood distributions of the Visuo-Tactile and Tactile-Only RTs are estimated, using as mean values the multiplication from the contrast matrixes of random and fixed effects, and the fixed and random estimates  $\mu_{Visuo-Tactile}\sim X\times visuo-tactile \beta_{1\ldots k}+Z \times u$  ${RT}_{Visuo-Tactile}\sim Normal(\mu_{Visuo-Tactile},1/sdscal)$  If ι = H_0,_ $\mu_{Tactile-Only}\sim X\times visuo-tactile \beta_{1\ldots k}+Z \times u$  If ι = H_1,_ $\mu_{Tactile-Only}\sim tactile-only \tau_{1}+Z \times u$  If ι = H_2,_ $\mu_{Tactile-Only}\sim tactile-only \tau_{1\ldots k}+Z \times u$  ${RT}_{Tactile-Only}\sim Normal(\mu_{Tactile-Only},1/sdscal)$ |
| --- | --- | --- |

### SM1.2 – Model for the analyses of Reaction Times for the Estimation of parameters

This model was used to analyse both Tactile-Only or Normalised Visuo-Tactile RTs to obtain estimates for the graphical representations.

It is a typical Multilevel Bayesian Linear model.

| 1  2  3  4  5  6  7  8  9  10  11  12  13  14  15  16  17  18  19  20  21  22  23  24  25  26  27  28  29  30  31  32  33  34  35  36  37  38  39 | model **{**  # Define priors for the variance-covariance matrix of the random effects  Omega.u **~** dwish**(**W**[**,**]**, dfwish**)**  Sigma.u **<-** inverse**(**Omega.u**[**,**])**    # Define hyperpriors for random effects  **for(**k **in** 1**:(**dfwish**-**1**)){**  xi_tau**[**k**]** **<-**xi_mu**[**k**]***sqrt**(**Sigma.u**[**k,k**])**  xi_mu**[**k**]** **~** dnorm**(**0, 0.001**)**  **}**    # Define correlations among random effects  **for(**r1 **in** 1**:(**dfwish**-**2**)){**  **for(**r2 **in** **(**r1**+**1**):(**dfwish**-**1**)){**  rho**[**r1,r2**]** **<-** Sigma.u**[**r1,r2**]/**sqrt**(**Sigma.u**[**r1,r1**]***Sigma.u**[**r2,r2**])**  **}**  **}**    # Define priors for the random effects  **for** **(** s **in** 1**:**Nsubjects **)** **{**  u**[**s,1**:(**dfwish**-**1**)]** **~** dmnorm**(**xi_mu**[**1**:(**dfwish**-**1**)]**,Omega.u**[**1**:(**dfwish**-**1**)**,1**:(**dfwish**-**1**)])**  **}**    # population parameters (fixed parameters)  **for** **(** j **in** 1**:**Nparameters **)** **{**  beta**[**j**]~** dnorm**(**0,1**/**s**[**j**])**    s**[**j**]** **~** dgamma**(**1,0.001**)**  **}**  sigma **~** dgamma**(**1,0.001**)**  #likelihood  **for** **(**i **in** 1**:**Ntotal**)** **{**  mu**[**i**]** **<-** inprod**(**XF **[**i,**]**,beta**)** **+** inprod**(**XR **[**i,**]**, u**[**subj **[**i**]**,**])**  RT **[**i**]** **~** dnorm**(**mu **[**i**]**,1**/**sdscal**)**  **}**  **}** | The variance-covariance matrix for the random effects comes from the Inverse of a Wishart distribution.  $\Omega\sim Wishart(diag(dfwish-1),dfwish )$  $\Sigma=\Omega^{-1}$  The coefficients of the random parameters are then estimated  $\xi_{1\ldots k}\sim Normal\left( 0,0.001 \right)$  ${sd\xi}_{1\ldots k}=\xi_{1\ldots k}\times\Sigma_{k,k}$  Next, also the correlation values between random parameters are computed  $\rho_{i,j}={\Sigma_{i,j}}/{\sqrt{\Sigma_{i,i}\times\Sigma_{j,j}}}$  Finally, the random effects for each participant is estimated  $u_{s,k}\sim MultiNormal(\xi_{1\ldots k}$,$\Omega_{k,k})$  Then, we estimate the fixed effects parameters for the RTs  $\sigma_{j}\sim Gamma(1,0.001)$  $\beta_{j}\sim Normal(0,1/{\sigma_{j}})$  Next, we estimate the variance of the Likelihood distribution  $\sigma\sim Gamma\left( 1,0.001 \right)$  Finally, the likelihood distributions of RTs are estimated, using as mean values the multiplication from the contrast matrixes of random and fixed effects, and the fixed and random estimates  $\mu\sim X\times\beta_{1\ldots k}+Z \times u$  $RT\sim Normal(\beta,1/sdscal)$ |
| --- | --- | --- |

### SM1.3 – Model for the analyses of Reaction Times for the Indicator Variable Selection

This model was used to analyse both Tactile-Only and Normalised Visuo-Tactile RTs to see which factors better explain the RTs by means of Indicator Variable Selection.

| 1  2  3  4  5  6  7  8  9  10  11  12  13  14  15  16  17  18  19  20  21  22  23  24  25  26  27  28  29  30  31  32  33  34  35  36  37  38  39  40  41  42  43  44  45  46  47  48  49  50  51  52  53  54  55  56  57  58  59  60  61  62  63  64  65  66  67  68  69  70  71  72  73  74  75  76  77  78  79  80  81  82  83  84 | model **{**  # Define priors for the IVS  **for(j** **in** 1**:nfactors){**  iot**[j]** **~** dbern**(**0.5**)**  **}**  iota <- c(iot[1],iot[1],iot[1],iot[1],  iot[2],iot[2],  iot[3],iot[3],  iot[1]*iot[2]*iot[4],  iot[1]*iot[2]*iot[4],  iot[1]*iot[2]*iot[4],  iot[1]*iot[2]*iot[4],  iot[1]*iot[2]*iot[4],  iot[1]*iot[2]*iot[4],  iot[1]*iot[2]*iot[4],  iot[1]*iot[2]*iot[4],  iot[1]*iot[3]*iot[5],  iot[1]*iot[3]*iot[5],  iot[1]*iot[3]*iot[5],  iot[1]*iot[3]*iot[5],  iot[1]*iot[3]*iot[5],  iot[1]*iot[3]*iot[5],  iot[1]*iot[3]*iot[5],  iot[1]*iot[3]*iot[5],  iot[2]*iot[3]*iot[6],  iot[2]*iot[3]*iot[6],  iot[2]*iot[3]*iot[6],  iot[2]*iot[3]*iot[6],  iot[1]*iot[2]*iot[3]*iot[4]*iot[5]*iot[6]*iot[7],  iot[1]*iot[2]*iot[3]*iot[4]*iot[5]*iot[6]*iot[7],  iot[1]*iot[2]*iot[3]*iot[4]*iot[5]*iot[6]*iot[7],  iot[1]*iot[2]*iot[3]*iot[4]*iot[5]*iot[6]*iot[7],  iot[1]*iot[2]*iot[3]*iot[4]*iot[5]*iot[6]*iot[7],  iot[1]*iot[2]*iot[3]*iot[4]*iot[5]*iot[6]*iot[7],  iot[1]*iot[2]*iot[3]*iot[4]*iot[5]*iot[6]*iot[7],  iot[1]*iot[2]*iot[3]*iot[4]*iot[5]*iot[6]*iot[7],  iot[1]*iot[2]*iot[3]*iot[4]*iot[5]*iot[6]*iot[7],  iot[1]*iot[2]*iot[3]*iot[4]*iot[5]*iot[6]*iot[7],  iot[1]*iot[2]*iot[3]*iot[4]*iot[5]*iot[6]*iot[7],  iot[1]*iot[2]*iot[3]*iot[4]*iot[5]*iot[6]*iot[7],  iot[1]*iot[2]*iot[3]*iot[4]*iot[5]*iot[6]*iot[7],  iot[1]*iot[2]*iot[3]*iot[4]*iot[5]*iot[6]*iot[7],  iot[1]*iot[2]*iot[3]*iot[4]*iot[5]*iot[6]*iot[7],  iot[1]*iot[2]*iot[3]*iot[4]*iot[5]*iot[6]*iot[7])  # Define priors for the variance-covariance matrix of the random effects  Omega.u **~** dwish**(**W**[**,**]**, dfwish**)**  Sigma.u **<-** inverse**(**Omega.u**[**,**])**    # Define hyperpriors for random effects  **for(**k **in** 1**:(**dfwish**-**1**)){**  xi_tau**[**k**]** **<-**xi_mu**[**k**]***sqrt**(**Sigma.u**[**k,k**])**  xi_mu**[**k**]** **~** dnorm**(**0, 0.001**)**  **}**    # Define correlations among random effects  **for(**r1 **in** 1**:(**dfwish**-**2**)){**  **for(**r2 **in** **(**r1**+**1**):(**dfwish**-**1**)){**  rho**[**r1,r2**]** **<-** Sigma.u**[**r1,r2**]/**sqrt**(**Sigma.u**[**r1,r1**]***Sigma.u**[**r2,r2**])**  **}**  **}**    # Define priors for the random effects  **for** **(** s **in** 1**:**Nsubjects **)** **{**  u**[**s,1**:(**dfwish**-**1**)]** **~** dmnorm**(**xi_mu**[**1**:(**dfwish**-**1**)]**,Omega.u**[**1**:(**dfwish**-**1**)**,1**:(**dfwish**-**1**)])**  **}**    # population parameters (fixed parameters)  **for** **(** j **in** 1**:**Nparameters **)** **{**  beta**[**j**]~** dnorm**(**0,1**/**s**[**j**])**    s**[**j**]** **~** dgamma**(**5,0.001**)**  **}**  sigma **~** dgamma**(**5,0.001**)**  #likelihood  **for** **(**i **in** 1**:**Ntotal**)** **{**  mu**[**i**]** **<-** inprod**(**XF **[**i,**]**,beta*c(1,iota**))** **+** inprod**(**XR **[**i,**]**, u**[**subj **[**i**]**,**])**  RT **[**i**]** **~** dnorm**(**mu **[**i**]**,1**/**sdscal**)**  **}**  **}** | Estimation of the ι hyper-hyperprior for each factor. It is distributed as a Bernoulli distribution, with equal probability for 0 and 1 outcomes.  $\iota\sim Bernoulli(0.5)$  Transformation of the ι hyper-hyperprior in the vector necessary to be multiplied with the β fixed effects.  The ι of the interaction between factors are multiplied for the ιs of the main effects, or lower interactions that are concerning that interaction. Therefore, if only one of them is equal to 0, the interaction will be equal to 0.  For example, the ι concerning the interaction Distance:Ligh.Loc. Location is multiplied by the ι of the main effect Distance, and by the ι of the main effect Ligh.Loc. Location. |
| --- | --- | --- |

## SM2 – Frequentist analyses

The frequentist analyses we used are ANOVAs, and as post-hoc tests we used Pairwise Welch t-tests corrected Holm-Bonferroni.

### SM2.1 – ANOVA on Tactile-Only Reaction Times

|  | num Df | den Df | MSE | F | pη^2^ | Pr(>F) |  |
| --- | --- | --- | --- | --- | --- | --- | --- |
| Distance | 4 | 156 | 5519.335 | 30.102 | 0.436 | 0.000 | *** |
| Tactile Location | 2 | 78 | 9016.118 | 20.668 | 0.346 | 0.000 | *** |
| Light Location | 2 | 78 | 4874.093 | 1.162 | 0.029 | 0.318 |  |
| Distance:Tactile Location | 8 | 312 | 3202.230 | 0.549 | 0.014 | 0.819 |  |
| Distance:Light Location | 8 | 312 | 2940.279 | 1.113 | 0.028 | 0.354 |  |
| Tactile:Light Location | 4 | 156 | 6109.481 | 1.813 | 0.044 | 0.129 |  |
| Distance:Tactile:Light Location | 16 | 624 | 3762.788 | 0.927 | 0.023 | 0.538 |  |

*Table SM2.1: Anova on the Tactile-Only RTs. num Df = Numerator degrees of freedom; den Df = Denominator degrees of freedom; MSE = Mean Squared Error; F = F value; pη^2^ = effect size; Pr(>F) = p-value*

As it is possible to observe, only two main effects are significant: the Distance factor and the Tactile Location factor.

Post-hoc tests on the Tactile Location factor showed that between Face and Foot, and between Face and Hand there are statistically significant differences (p < 0.01), while the difference between Hand and Foot is not statistically significant (p = 0.07).

Their mean (SD) RTs are: Face 356.128 (127.154), Hand 377.862 (129.308) and Foot 391.026 (121.858).

A-posteriori tests on the Distance factor showed that all comparisons are statistical significant, with the exception of the comparison between D1 and D5, that has a strong trend towards the statistical significance (p = 0.056). Means and standard deviations are: D1 = 370.722 (124.072); D2 = 349.182 (114.766); D3 = 402.959 (135.37); D4 = 362.35 (129.705); D5 = 389.813 (122.986).

### SM2.2 – ANOVA on Normalized Visuo-Tactile Reaction Times

|  | num Df | den Df | MSE | F | pη^2^ | Pr(>F) |  |
| --- | --- | --- | --- | --- | --- | --- | --- |
| Distance | 4 | 156 | 15166.808 | 50.949 | 0.566 | 0.000 | *** |
| Tactile Location | 2 | 78 | 11684.321 | 0.654 | 0.017 | 0.523 |  |
| Light Location | 2 | 78 | 9113.472 | 0.281 | 0.007 | 0.756 |  |
| Distance:Tactile Location | 8 | 312 | 8972.949 | 1.684 | 0.041 | 0.101 |  |
| Distance:Light Location | 8 | 312 | 7336.498 | 2.261 | 0.055 | 0.023 | * |
| Tactile:Light Location | 4 | 156 | 9969.806 | 2.438 | 0.059 | 0.049 | * |
| Distance:Tactile:Light Location | 16 | 624 | 8280.455 | 0.739 | 0.019 | 0.754 |  |

*Table SM2.2: Anova on the normalized Visuo-Tactile RTs. num Df = Numerator degrees of freedom; den Df = Denominator degrees of freedom; MSE = Mean Squared Error; F = F value; pη^2^ = effect size; Pr(>F) = p-value*

ANOVA results show a main effect of Distance, showing a general PPS effect, and the interaction Distance:Light Location showing that the PPS effect is mainly linked to the location of the Visual stimuli, and the interaction between Tactile and Light Location that, because it is not in interaction with Distance, it is not a PPS effect.

Post-hoc tests on Distance showed that all levels were statistically different from the others (all ps < 0.001). D1 = -26.2 (68.965); D2 = -52.786 (72.04); D3 = 5.776 (118.128); D4 = 62.909 (95.243); D5 = 35.317 (123.457).

Post-hoc tests on the Tactile:Light Location interaction showed that no comparison survived to the post-hoc correction.

Post-hoc tests on the Distance:Light Location interaction showed that the first significant difference between Distances when the Light Location is the Face is between D2 and D3 (p = 0.043); when the Light Location is the Hand is between D1 and D2 (p < 0.001); and when the Light Location is the Foot the difference is between D3 and D4 (p = 0.002).

| **Distance** | **Face** | **Hand** | **Foot** |
| --- | --- | --- | --- |
| *D1* | -32.527 (66.277) | -15.012 (55.238) | -31.06 (81.941) |
| *D2* | -45.306 (56.568) | -69.358 (92.183) | -43.693 (59.517) |
| *D3* | -0.511 (73.662) | 18.951 (177.603) | -1.111 (69.753) |
| *D4* | 64.459 (75.3) | 71.625 (73.11) | 52.644 (127.144) |
| *D5* | 41.575 (132.773) | 27.541 (66.09) | 36.837 (154.537) |

*Table SM2.2.1: mean and standard deviation for each Light Location and Distance.*
